# Metacarpal tuberculosis with Nocardia infection: A case report

**DOI:** 10.1097/MD.0000000000018804

**Published:** 2020-03-13

**Authors:** Ruohui Tang, Jing Yang, Huayu Liu, Kai Zhou, Jun Fei

**Affiliations:** a Emergency Department of Daping Hospital; b Trauma center of Daping Hospital, Third Military Medical University (Army Medical University); c Department of Orthopedics, the First Affiliated Hospital of Chongqing Medical University; d State Key Laboratory of Trauma, Burns and Combined Injury, Third Military Medical University (Army Medical University), Chongqing, China.

**Keywords:** bone tuberculosis, masquelet technique, nocardia

## Abstract

**Introduction:**

Isolated metacarpal tuberculosis is rare in orthopedic surgery. In the case of poor efficacy of traditional treatment methods, such as debridement surgery and anti-tuberculosis treatment, it is necessary to consider whether there is a special type of infection. We describe a case of metacarpal tuberculosis with *Nocardia* infection in a patient.

**Patient concerns:**

A 65-year-old male patient who suffered from pain and dysfunction lasted for 6 years.

**Diagnoses:**

Confirmation of the diagnosis was finally achieved by isolation of *M tuberculosis* and *Nocardia actinomycetes* from bone specimens.

**Interventions:**

The patient underwent debridement surgery, Masquelet technique was used during the operation, and oral antibiotics were combined after surgery.

**Outcomes:**

Bone graft surgery was performed 6 weeks after the first surgery. We followed up on bone healing at 1 and 3 months postoperatively.

**Conclusion:**

Tissue-specific necrosis usually occurs in particular types of infections such as tuberculosis, which limits the spread of antibiotics. Masquelet technique seems to bring new options to solve this problem. The performance of *Nocardia* infection is similar to that of tuberculosis infection, so it is difficult to identify clinically. Therefore, for cases where tuberculosis is suspected, and anti-tuberculosis treatment is ineffective, the possibility of *Nocardia* infection needs to be considered.

## 1 Introduction

Tuberculosis infections that occur in the bone account for about 10% of the body.^[1]^ The spine is the most common site of involvement, accounting for about 3% of bone tuberculosis.^[2]^ Bone tuberculosis involving the metacarpal is rare. Mixed infections of other pathogens are often combined with tuberculosis infection. As far as we know, this is the first report of bone tuberculosis combined with *Nocardia* infection.

## 2 Case introduction

**Figure 1:**
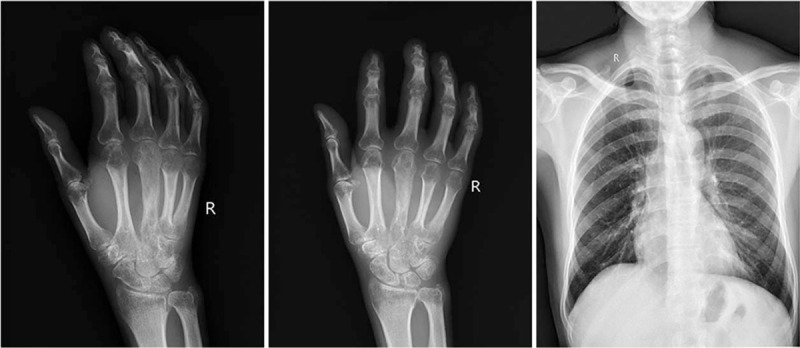
X-ray image before surgery.

A 65-year-old male patient has no history of the application of hormones or immunosuppressive agents. Two years ago, he underwent debridement surgery in other hospitals and treated with oral antibiotics for 7 weeks. He denied recent symptoms of fever, trauma or physical discomfort. The patient's vital signs were normal at admission. Laboratory examinations and imaging studies were performed after admission. Among them: white blood cell 4.92 × 10^9^/L, Lymphocyte% 17.7%, Monocyte% 10.3%, Neutrophil% 71.3%, C-reactive protein-3 48.3 mg/L, erythrocyte sedimentation rate 26 mm/h. X-ray images indicate bone destruction and reactive hyperplasia. There was no obvious abnormality in the chest X-ray image (Fig. 1).

**Figure 2:**
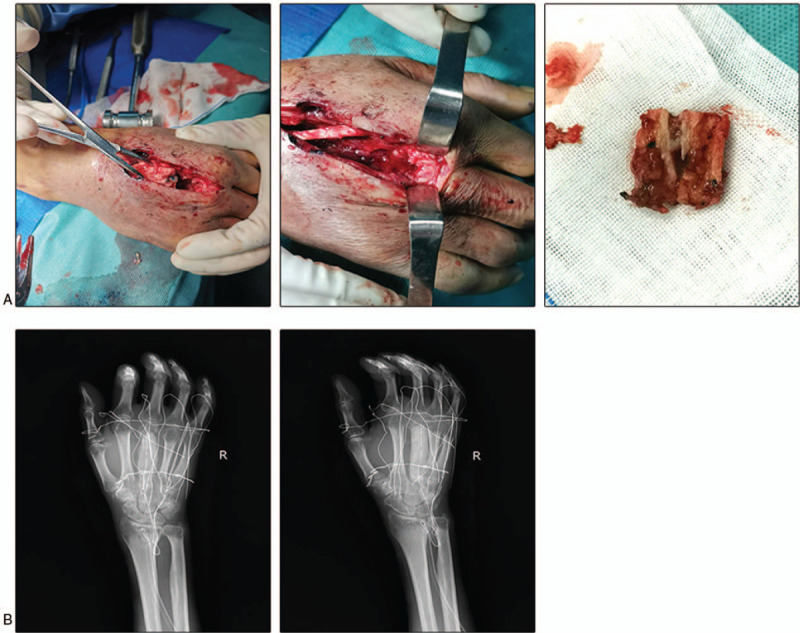
(A) Images during operation. The lesions around the metacarpal bones are wrapped with cheese-like tissue, combined with severe bone destruction. (B) X-ray image after surgery.

**Figure 3:**
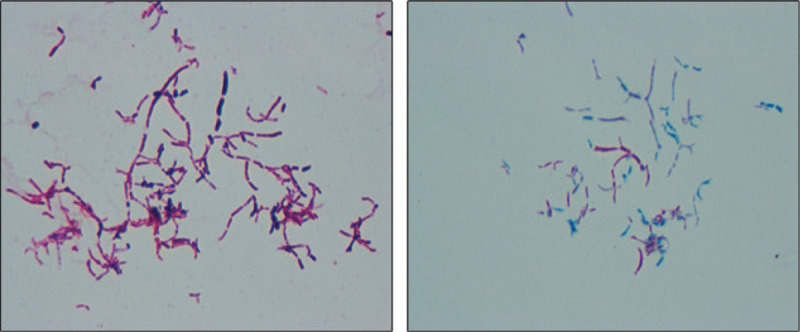
Gram-stained image and acid-resistant stained image (weakly positive).

**Figure 4:**
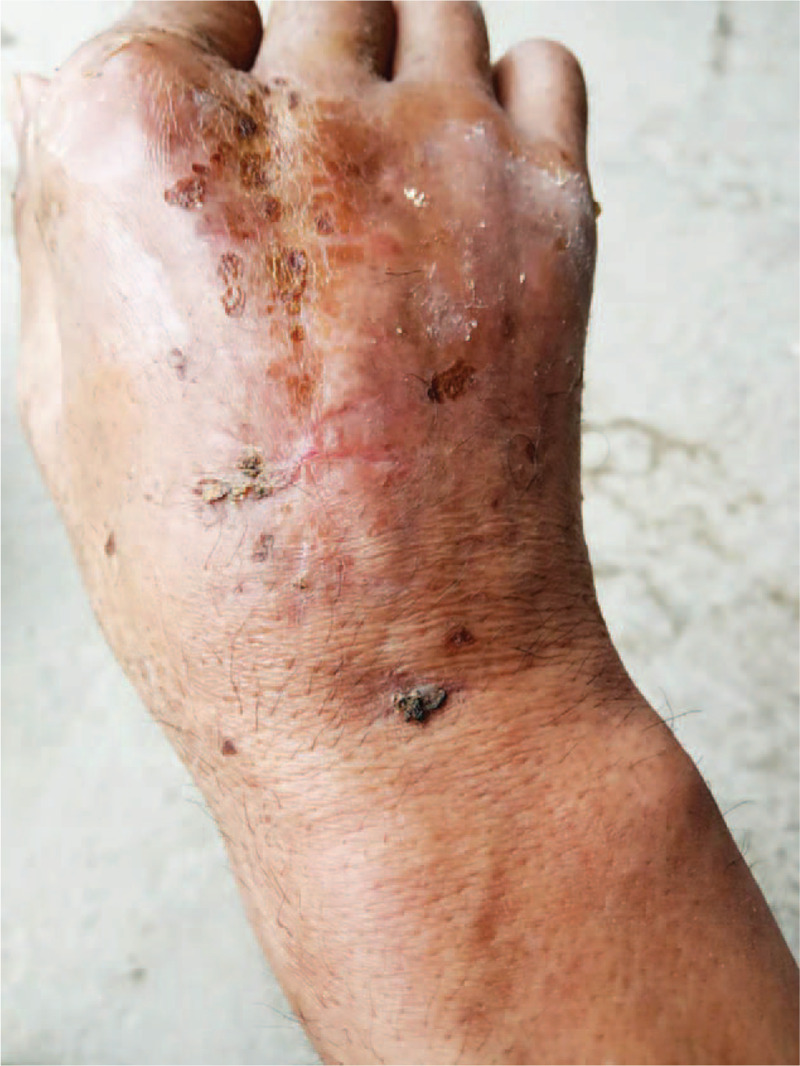
Image of the patient after 1 mo of follow-up. The wound on the hand has healed.

**Figure 5:**
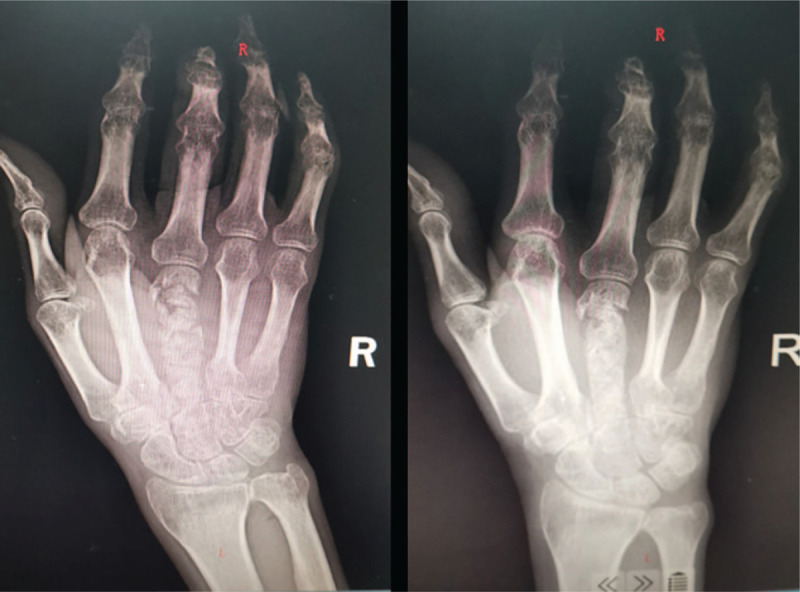
X-ray image during follow-up. left (1 mo), right (3 mo).

We injected methylene blue from the wound to mark the boundaries of necrotic tissue. Combined with the medical history, intraoperative performance and radiological evidence, we suspect that it is a tuberculosis infection. Surgery consists primarily of removing necrotic tissue and bone cement loaded with antibiotics to fill the lesion. Streptomycin was used in operation, and the ratio of antibiotic to bone cement was 1:6.^[3]^ Gypsum external fixation brake was given after the operation (Fig. 2). The patient started oral anti-tuberculosis drugs after surgery, including rifampicin (0.5 g/d), isoniazid (0.3 g/d), and ethambutol (1.2 g/d). Improved Roche’ s (L-J) culture method showed *M tuberculosis*. Pathological examination showed that fibroplasia and inflammatory granulation tissue were formed in the bone tissue, and microabscess formation was observed locally. Wound healing was found to be less than satisfactory during follow-up. Sulfamethoxazole (1.2 g/d) was added to the treatment regimen because of the bacteriological detection of *Nocardia.* The colony morphology under the microscope is Gram-positive, branched, and the hyphae can be entangled into a mass to form particles similar to actinomycetes, and the acid-fast staining is weakly positive. (Fig. 3) The result of mass spectrometry identification was *Nocardia. Yamanashi*. Bone graft surgery was performed 6 weeks after the first surgery. At 1 month of follow-up, the patient had no clinical symptoms and the wound healed well (Fig. 4). Sulfonamide treatment lasted for 6 months.^[4]^ Liver and kidney function were dynamically reviewed during follow-up to adjust the drug dose. X-ray examination was performed 1 month and 3 months after the bone graft operation, and the bone gap was gradually blurred. (Fig. 5)

## 3 Discussion

Tuberculosis infection remains a significant regional public health problem in the world. Atypical clinical manifestations and disease progression often make tuberculosis infection difficult to diagnose accurately. Although the spine is a common site of bone tuberculosis, tuberculosis infection may occur in all bones and joints.^[5]^ Therefore, the diagnosis of bone tuberculosis is often difficult and has a high rate of misdiagnosis. Improved Roche’ s (L-J) culture method is still the gold standard for the clinical diagnosis of tuberculosis. The diagnosis and treatment of mixed infections is a huge clinical challenge. HIV infects other cells, including macrophages, dendritic cells, neutrophils, and T cell interactions, which may affect susceptibility to tuberculosis infection, disease progression and severity.^[6]^ In addition, the occurrence of intracellular bacterial infection such as Salmonella, Listeria or Chlamydia is closely related to the host response caused by Mycobacterium tuberculosis.^[7]^ The development of new testing techniques has also provided new options for the diagnosis of tuberculosis infection, such as mycobacterial-gene sequencing.^[8]^

Surgery and medicine are indispensable for the treatment of bone tuberculosis. Clinically available anti-tuberculosis drugs include isoniazid, rifampicin, ethambutol, streptomycin, and so on. The development of new anti-tuberculosis drugs is difficult. Bedaquiline is a new drug recently approved by the US FDA for tuberculosis treatment, and its efficacy depends on a wider range of applications to evaluate.^[9]^ In addition to surgery and the application of anti-tuberculosis drugs, the treatment of bone defects is also a challenge. In 2000, Masquelet et al used polymethylmethacrylate bone cement to induce the formation of periosteal-like structure in the defect, and then filled the area with autologous cancellous bone graft, and achieved satisfactory results.^[10]^ It is currently used in the repair of bone tumors and chronic osteomyelitis. In recent years, many scholars have gradually realized the superiority of this technology, and have carried out a lot of useful explorations on the bone grafting methods and fracture fixation methods in operation, which promoted the development of this technology.^[11]^ We performed Masquelet technique in this particular type of infection treatment. The part of the clinical research is ongoing.

Tuberculosis infection occurs mostly in people with low immunity. Such infections are often not caused by a single pathogen.^[6]^*Nocardia* is also an opportunistic pathogen that causes disseminated infections in the lungs and skin.^[12]^ In recent years, with the widespread use of immunosuppressants and antibiotics, the incidence of *Nocardia* infection is on the rise.^[13]^ Studies have reported that the mortality rate of *Nocardia* lung infection in immunosuppressed people is 40%.^[14]^ The patient we reported lacked diagnostic evidence for *Nocardia* infection in the lungs. Report of primary *Nocardia* infection in bone tissue is also rare. The main pathological manifestation of *Nocardia* infection is suppurative inflammation with abscess formation. The symptoms of lung infection are not specific, including fever, cough, sputum, and so on. *Nocardia* infection in skin soft tissue mainly manifested as subcutaneous abscess, nodules, ulceration, and sinus formation. The effectiveness of sulfa drugs for *Nocardia* infection was confirmed.^[15]^ Besides, tetracyclines, streptomycin, and amikacin have also been reported to be effective against *Nocardia* infection.^[16]^ Long-term treatment is a routine choice, but the duration of medication is uncertain.^[17]^ To treat a particular type of infection, a more extended period of medication is often chosen, accompanied by an increase in the incidence of drug-resistant bacteria and adverse drug reactions. Tissue-specific necrosis usually occurs in particular types of infections such as tuberculosis, which limits the spread of antibiotics. Masquelet technique seems to bring new options to solve this problem. The performance of *Nocardia* infection is similar to that of tuberculosis infection, so it is difficult to identify clinically. Therefore, for cases where tuberculosis is suspected, and anti-tuberculosis treatment is ineffective, the possibility of *Nocardia* infection needs to be considered.

In summary, the diagnosis and treatment of bone infections caused by specific types of pathogens require more attention. The exploration of new treatment methods is of great significance for shortening the use of antibiotics and improving the efficacy.

## Author contributions

**Conceptualization:** Ruohui Tang, Jun Fei.

**Data curation:** Jing Yang.

**Methodology:** Ruohui Tang, Huayu Liu.

**Writing – original draft:** Ruohui Tang.

**Writing – review and editing:** Jing Yang, Kai Zhou, Jun Fei.
